# On geographic barriers and Pleistocene glaciations: Tracing the diversification of the Russet-crowned Warbler (*Myiothlypis coronata*) along the Andes

**DOI:** 10.1371/journal.pone.0191598

**Published:** 2018-03-09

**Authors:** David A. Prieto-Torres, Andrés M. Cuervo, Elisa Bonaccorso

**Affiliations:** 1 Master Oficial en Biodiversidad en Áreas Tropicales y su Conservación. Universidad Internacional Menéndez Pelayo, Madrid, España; 2 Eje BioCiencias, Centro de Modelado Científico de la Universidad del Zulia (CMC-LUZ), Facultad Experimental de Ciencias. Calle 65 con Av. Universidad, sector Grado de Oro, Estado Zulia, Maracaibo, Venezuela; 3 Department of Biological Sciences and Museum of Natural Science, Louisiana State University, Baton Rouge, Louisiana, United States of America; 4 Instituto BIOSFERA and Colegio de Ciencias Biológicas y Ambientales, Universidad San Francisco de Quito, vía Interoceánica y Diego de Robles, Quito, Ecuador; 5 Centro de Investigación de la Biodiversidad y Cambio Climático, Universidad Tecnológica Indoamérica, Machala y Sabanilla, Quito, Ecuador; Sichuan University, CHINA

## Abstract

We studied the phylogeography and plumage variation of the Russet-crowned Warbler (*Myiothlypis coronata*), from Venezuela to Bolivia, with focus on populations from Ecuador and northern Peru. We analyzed sequences of mitochondrial and nuclear genes, geographic distributions, as well as photographs of specimens deposited at museum collections. Phylogenetic analyses identified three major lineages formed by populations from: Venezuela and Colombia (*M*. *c*. *regulus*), Ecuador and northern Peru (*M*. *elata*, *M*. *castaneiceps*, *M*. *orientalis*, *M*. *c*. *chapmani*), and central Peru and Bolivia (*M*. *c*. *coronata*). We found further population structure within *M*. *c*. *regulus* and *M*. *c*. *coronata*, and population structure and complexity of plumage variation within the Ecuador-northern Peru lineage. Time-calibrated trees estimated that most intraspecific variation originated during the Pleistocene; however, this pattern may not be attributed to an increase in diversification rate during that period. We discuss these results in the context of the importance of geographic-ecological barriers in promoting lineage diversification along the Andes and put forward a preliminary taxonomic proposal for major lineages identified in this study.

## Introduction

Our collective understanding of evolutionary patterns of Andean species has advanced rapidly in the last decade. Thanks to a solid body of research, geographic-ecological barriers have been identified as important drivers of evolutionary change [1–4]. After causing initial limitation or cessation of gene flow, these features may provide fertile grounds for other biological forces, such as natural and sexual selection [5, 6], or stochastic processes [7, 8]. Thus, studying population evolution across these features provides opportunities for witnessing speciation at its initial stages [9], and has potential to allow identification of evolutionarily significant units for conservation [10]. The latter is likely to be useful in helping Andean countries to effectively focus limited resources on preserving species and genetic diversity, as well as long-term evolutionary processes [11].

The Russet-crowned Warbler, *Myiothlypis coronata*, is an ideal organism for studying evolutionary diversification along the Andes. It is a humid mountain forest species with a fragmented distribution from western Venezuela south to central Bolivia [12], currently represented by eight subspecies, which vary mainly in ventral coloration. *Myiothlypis coronata regulus* (western Venezuela to Colombian Andes) and *M*. *c*. *elata* (extreme southwestern Colombia to southwestern Ecuador), have gray throat, and yellow breast and belly; *M*. *c*. *castaneiceps* (southwest Ecuador and northern Peru) and *M*. *c*. *chapmani* (east slope of west Andes in northern Peru), have greyish-white throat, breast, and belly; *M*. *c*. *orientalis* (east slope of east Andes of Ecuador), has been described as an intermediate of yellow and gray forms; *M*. *c*. *inaequalis* (central Andes in northern Peru), *M*. *c*. *coronata* (E Andes from central Peru to western Bolivia), and *M*. *c*. *notia* (east Andes in central Bolivia) also have yellow breast and belly [12, 13]. Differences within subspecies in the “yellow” and “grey” groups are given by minor changes in size, and color tone in crown and dorsum (see [12]). Also, in Ecuador, in the extreme southeast (Cordillera del Cóndor and Cordillera de las Lagunillas), birds have been described as morphologically intermediate between *M*. *c*. *orientalis* and *M*. *c*. *chapmani* [13].

Herein, we aim to understand the evolutionary history of *Myiothlypis coronata*, based on molecular phylogenetic analyses of samples from Venezuela to Bolivia. First, we explore whether plumage differentiation among subspecies of the Russet-crowned Warbler is coupled with genetic differentiation, and if genetic differentiation may be attributed to the presence of putative geographic-ecological barriers to dispersal. Second, given previous studies pointing to the importance of climatic oscillations of the Pleistocene in the evolution of Andean organisms [14–17], we ask whether most lineages within *M*. *coronata* evolved during that period. More specifically, we test if such pattern may be associated to evolutionary changes in response to Pleistocene climate cycles; under this hypothesis, we would expect an increase in lineage diversification rates during that period. Third, we refine our phylogeographic analyses along the Ecuadorian and north Peruvian Andes, where further complexity of coloration and distribution patterns might be resulting from incipient population divergence. To that end, we analyzed museum specimens from this region in detail, and assessed if genetic variation couples with geographic distribution and phenotypic differentiation among populations. With this information, we discuss how time and topography might have played a role in shaping distribution of populations of *M*. *coronata*.

## Materials and methods

Tissue samples of *Myiothylpis coronata* were obtained from museum collections (see Acknowledgments) and through our fieldwork in Colombia, Ecuador, and Venezuela. Analyses were mainly based on the mitochondrial gene NADH dehydrogenase Subunit 2 (ND2: 1041 base pairs [bp]) for 153 individuals distributed from Venezuela to Bolivia, plus sequences for *M*. *cinereicollis*, *M*. *conspicillatus*, *M*. *chrysogaster*, and for two additional outgroup species, *M*. *bivittatus* and *M*. *nigrocristatus*, from Lovette et al. [18] (S1 Table). For the three subspecies found in Ecuador (*M*. *c*. *elata*, *M*. *c*. *orientalis*, and *M*. *c*. *castaneiceps*), we also analyzed partial sequences of cytochrome b (cytb: 996 bp) for 42 individuals, and complete sequences of the transforming growth factor beta 2, intron 5 (TGFß2.5: 537 bp) for 17 individuals.

Genomic DNA was isolated from muscle tissue or blood with an extraction protocol based on protein precipitation with guanidinine thiocyanate, followed by DNA precipitation with isopropanol. Amplification by PCR was completed using the following primer pairs: L5219 or L5143, and H6313, for ND2 [19]; L14990 [20] and H16065 (T. Birt; GTCTTCAGTTTTTGGTTTACAAGAC), for cytb; and, TGFb2.5F and TGF2.6R, for TGFß2.5 [21]. Mitochondrial genes were amplified using a standard PCR protocol (i.e. 94°C/5min; 35 cycles of 93°C/1min, 52°C/1min, 72°C/2min; and 72°C/10min), whereas the TGFß2.5 was amplified using a touchdown protocol (i.e. 94°C⁄3 min; 5 cycles of 94°C⁄30 s, 60°C⁄30 s, 72°C⁄40 s; 5 cycles of 94°C⁄30 s, 56°C⁄30 s, 72°C⁄40; 35 cycles of 96°C⁄30 s, 52°C⁄30 s, 72°C⁄40 s; and 72°C⁄10 min). Amplification products were visualized in 2% agarose, unincorporated primers and dNTPs degraded using ExoSap-it (Affymetrix-USB), and sequencing reactions conducted at Macrogen Inc. (Korea), using PCR primers. Data from heavy and light strands were assembled in Geneious 5.4 [22]. Sequences were aligned in Clustal X2 [23], and were inspected and translated into proteins in Mesquite ver. 3.2 [24].

Phylogenetic trees were obtained using maximum likelihood (ML) and Bayesian analyses. We first used PartitionFinder2 [25] to determine the most appropriate partition scheme for all datasets (ND2, ND2 + cytb “Ecuador”, ND2 + cytb + TGFß2.5 “Ecuador”), with branch lengths linked, all models tested under the AIC, and “greedy” algorithm. Maximum likelihood trees were estimated using GARLI 2.0 [26], running 20 independent analyses with default settings. Bootstrap support (1000 pseudo replicates), was assessed running one replicate per search. Bayesian analyses were conducted in MrBayes 3.2 [27], using a random starting tree, with four simultaneous Markov chains run for 10,000,000 generations, sampling every 1000 trees, discarding the first 20% of trees as burn-in, and combining the remaining trees into a 50% majority-rule consensus tree.

To explore the timeframe for the evolution of different lineages within *Myiothlypis coronata*, we performed a time-calibrated phylogenetic analysis over the ND2 dataset, in BEAST 1.7.4 [28]. *Myothlypis fraseri* was used as outgroup, constraining *M*. *coronata* to be monophyletic. Analyses were run under the GTR + I + G substitution model, log-normal uncorrelated relaxed clock (0.0125 substitutions/site/Myr; [29]; Euclidian standard variation = 0.1), Yule speciation tree prior, running for 50 million generations, and sampling every 1000 generations. After checking for stationarity in Tracer [30] (all ESS over 200), we discarded the first 10,000 trees as burn-in and obtained a maximum clade probability tree with the remaining trees.

To explore the effect of Pleistocene climatic events on the evolution of *Myiothlypis coronata*, we tested for deviations from a constant diversification rate. Here, we assumed that genetically differentiated populations identified during phylogenetic analyses represented discrete evolutionary lineages. We re-ran the BEAST analysis including only one representative sample per lineage, using the same settings from the previous analysis, but running the analysis for only 10 million generations (2000 trees burn in). Then, we tested which of two constant rate models, a Yule pure-birth [31] or a birth-death process [32] best explained our BEAST tree, using Phytools 0.6–44 [33] as implemented in R 3.4.3 [34]. This comparison was based on both the likelihood ratio test and the Akaike Information Criterion (AIC). Then, we obtained a lineages through time (LTT) plot and applied the gamma (γ) test [35], which allows detecting changes in diversification rate. We also simulated 100 random trees assuming the appropriate speciation model, with the same duration and resulting in the same number of lineages as the empirical tree, and obtained LTT plots for those trees.

Population comparisons were performed as follows. Uncorrected and ML-corrected genetic pairwise distances were calculated in PAUP v.4.0a109 [36]; migration rates between adjacent lineages were estimated with MDiv [37], using the infinite sites model, maximum value for scaled migration rate = 10, scaled divergence time = 5, steps = 5 million, and burn-in = 500,000. In DNAsp [38] we calculated indices of molecular diversity and estimated deviations from neutrality (Tajimas' D and Fu's Fs) of ND2 sequences across lineages. Finally, in TCS 1.21 [39], we obtained a statistical parsimony network among *M*. *coronata* populations from Ecuador and northern Peru.

Plumage analysis of Ecuadorian populations (*Myiothlypis coronata elata*, *M*. *c*. *orientalis*, and *M*. *c*. *castaneiceps*) was based on examination of 40 specimens from ornithological collections (S1 Table). We took photographs of the venter, dorsum, and crown in RAW format using a gray background surface, and illumination with white lighting (4,500–5,500 K) with flash and diffuser. Photographs were analyzed in Photoshop CS6 for standardized comparisons. We also analyzed photographs of specimens of *M*. *c*. *elata*, *M*. *c*. *orientalis*, and alleged *M*. *c*. *chapmani* > *orientalis* deposited at the Academy of Natural Sciences of Philadelphia, and specimens identified as *M*. *c*. *castaneiceps* and *M*. *c*. *chapmani*, deposited at the Louisiana State University Museum of Natural Science. Although these later photographs were not taken under the same standardized conditions as those applied to Ecuadorian specimens, they were useful in widening the geographic scope of the analysis.

## Results

**Fig 1:**
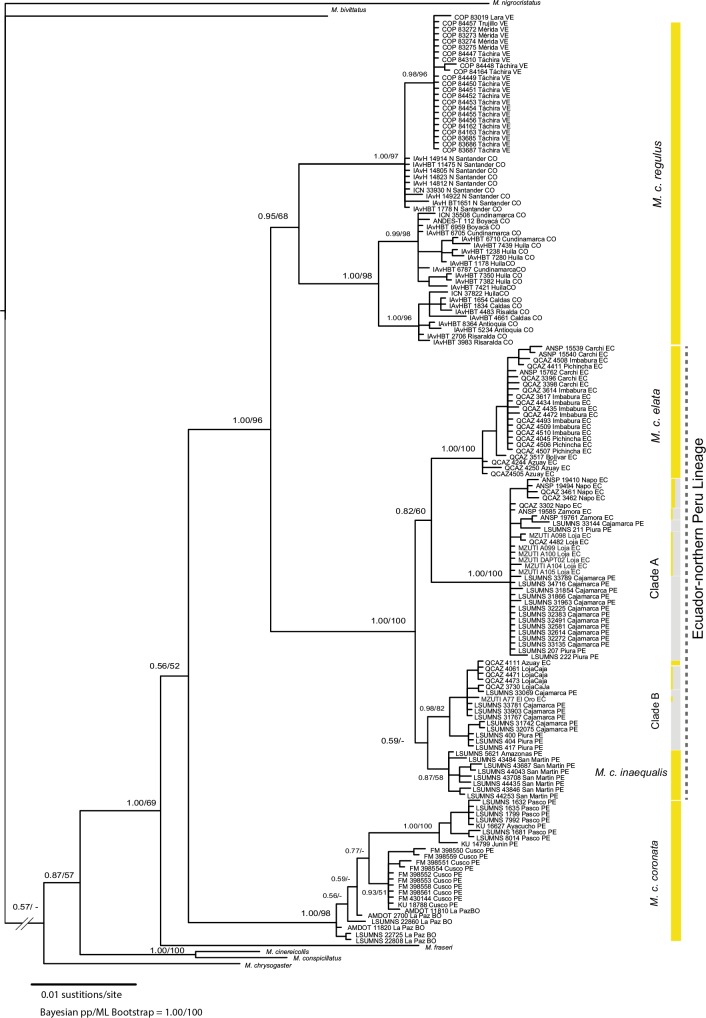
Bayesian tree based on the analysis of ND2, showing the correspondence of phylogroups and recognized subspecies. Bayesian probabilities and ML bootstrap support are provided next to major nodes. Analyses were run with a partition by codon, using the following models: HKY + I + G for 1st codon, TRN + I for 2nd codon, and TRN + G for 3rd codon. Color bars indicate an approximation to the dominant ventral plumage colors per sample.

Both Bayesian and ML analyses of ND2 estimated the same tree topology, showing *Myiothlypis fraseri* and *M*. *coronata* as sister species, and recovering three major lineages (Fig 1). The first lineage consists of samples of *M*. *c*. *coronata* (central Peru-Bolivia), and is sister to a group formed by all other populations of *M*. *coronata*. This later group is further divided into two lineages: one containing samples of *M*. *c*. *regulus* (Venezuela-Colombia), and another of samples from Ecuador and northern Peru (hereafter, Ecuador-northern Peru). The Ecuador-northern Peru lineage splits further into four clades: (1) *M*. *c*. *elata* (western Ecuador south to Azuay); (2) clade A (*M*. *c*. *orientalis* [eastern Ecuador, Napo]; *M*. *c*. *chapmani>orientalis* [Cordillera del Cóndor], and samples from southern Ecuador [Loja] and northern Peru [Cajamarca]); (3) clade B (one *M*. *c*. *elata* [Azuay], one *M*. *c*. *castaneiceps* [El Oro], and samples from southern Ecuador [Loja] and northwest Peru [Piura and Cajamarca]); and (4) *M*. *c*. *inaequalis* (Amazonas and San Martín). Sample details and GenBank Accession numbers are available in S1 Table.

Combined mitochondrial (ND2 + cytb) and mitochondrial-nuclear trees for the Ecuadorian samples did not provide additional information on topology. Both recovered three phylogroups within Ecuador (*M*. *c*. *elata*, clade A, and clade B), but did not resolve the relationships among them. Variation in the nuclear gene TGFß2.5 was restricted to only nine nucleotide substitutions (four autapomorphic) and a six base-pair indel in *M*. *fraseri*, providing no resolution regarding relationships among phylogroups. Sequences of cytb and TGFß2.5 were deposited on Genbank (Accession numbers MG721366– MG721407, and MG721408–MG721423, respectively).

**Fig 2:**
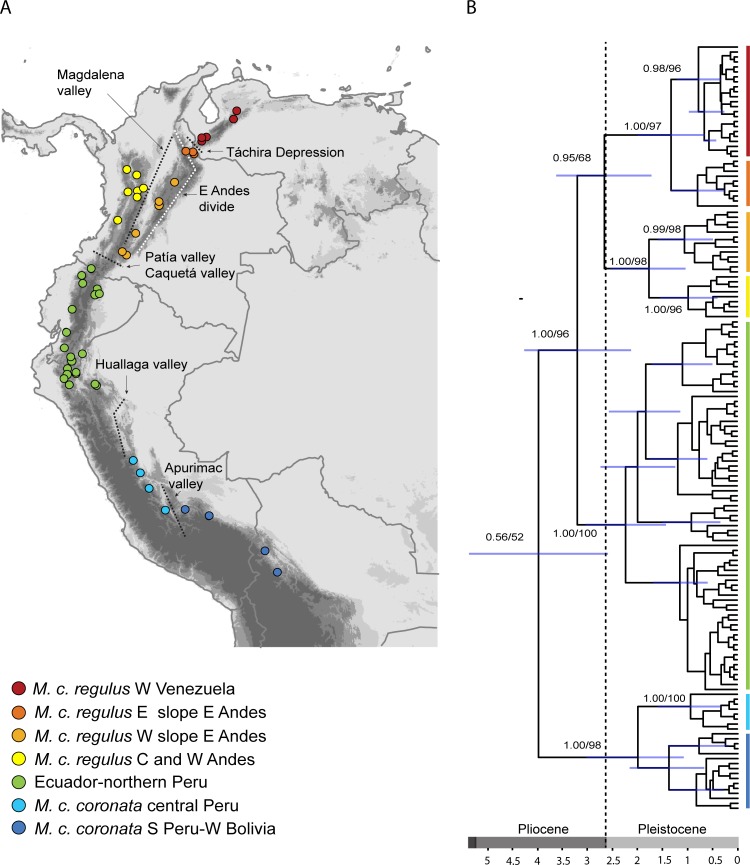
Phylogeography of *Myiothlypis coronata*. A. Geographic location of samples analyzed, as well as geographic breakpoints between populations. B. BEAST maximum clade credibility tree showing geographic location of major clades; Bayesian probabilities and bootstrap support (derived from the ML and Bayesian analyses) are provided for each phylogroup.

**Fig 3:**
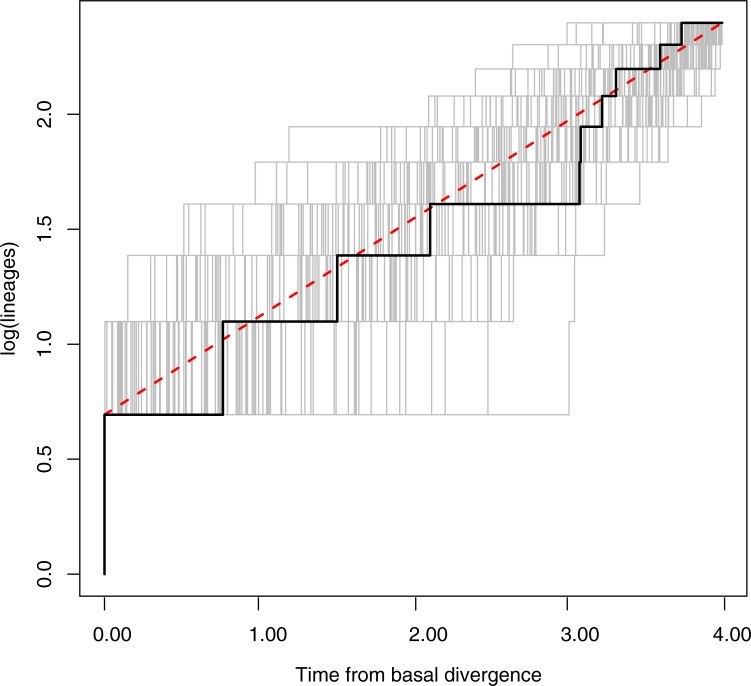
Lineages through time plot derived from the BEAST three of *Myiothlypis coronata* and 100 trees simulated under the Yule pure-birth model. Black line, LTT of *Myiothlypis coronata*; gray lines, LTT of simulated trees; red-dotted line, expectation of a constant rate. Number of lineages is presented in logarithmic scale.

Divergence-time estimation suggests that *M*. *coronata* originated in the late Miocene or in the Pliocene (95% Highest Posterior Density [HPD] = 5.4–2.6 million years), and that most genetic and morphological diversification at the population level occurred within the late Pliocene and the Pleistocene (Fig 2). The likelihood-ratio test could not reject the Yule pure-birth model over the more complex birth-death model (log likelihood 48.526 vs. 48.549; chi-squared = 0.0456; p = 0.839), and the AIC (AIC 0.727 vs. 0.2735) showed that the majority of weight of evidence favors the Yule model. The LTT plot was virtually linear (Fig 3) and the gamma (γ) statistic for the tree under the Yule model was 0.1243 (p = 0.9011), which is close to zero, indicating either constant diversification rate or constant extinction rate with decreasing speciation [40]. However, this later possibility is not supported by our LTT plot.

**Fig 4:**
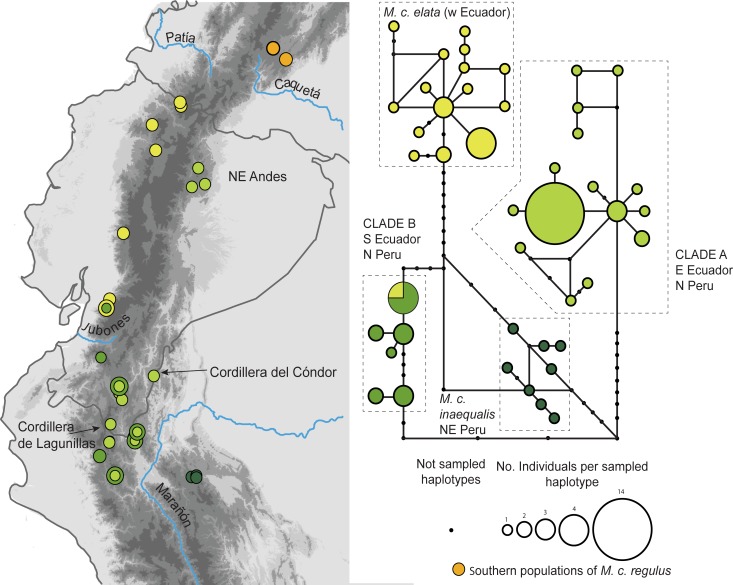
Statistical parsimony network of the 75 samples in the Ecuador-northern Peru lineage, and geographic location of samples.

The statistical parsimony network of 75 sequences of ND2 (Fig 4) recovered 44 different haplotypes (33 singletons) of *M*. *coronata* from Ecuador-northern Peru, which divided into four main networks that correspond to the same clades found in phylogenetic analyses: (1) west Ecuador (*M*. *c*. *elata*): (2) east Ecuador and northern Peru (clade A; *M*. *c*. *orientalis*, *M*. *c*. *chapmani>orientalis*, Loja, and Cajamarca); (3) southwest Ecuador and northern Peru (clade B; *M*. *c*. *castaneiceps*, Loja, Cajamarca, and Piura); and (4) north eastern Peru (*M*. *c*. *inaequalis*: Amazonas and San Martín). Samples from the same localities (Cajanuma, [Loja]; Quebrada Lanchal, Cordillera del Cóndor, and San José de Lourdes [Cajamarca]) belong to either clade A or B. Again, one sample of *M*. *c*. *elata* is located within clade B.

Uncorrected and ML-corrected genetic distances were higher between samples of the Ecuador-northern Peru lineage and *M*. *c*. *coronata*, and samples of *M*. *regulus* and *M*. *coronata* (Table 1). As expected, migration-rate analyses (S1 Fig) estimated small numbers of migrants per generation between *M*. *c*. *regulus* (Colombia-Venezuela) vs. Ecuador-northern Peru, and Ecuador-northern Peru vs. *M*. *c*. *coronata* (maximum likelihood estimate of number of migrants, MLE [Nm] = 0.02 in both cases). Within the Ecuador-northern Peru lineage, numbers of migrants were, as follows: *M*. *c*. *elata* vs. clade A, MLE [Nm] = 0.02; *M*. *c*. *elata* vs. clade B, MLE [Nm] = 0.16; clade A vs. clade B, MLE [Nm] = 0.1; clade A vs. *M*. *c*. *inaequalis*, MLE [Nm] = 0.02; and, clade B vs. *M*. *c*. *inaequalis*, MLE [Nm] = 0.04. Tajimas' D and Fu's Fs statistics found no deviations from neutrality with one exception: Tajimas' D for *M*. *c*. *elata*, was significant because the sample in clade B introduced a high number of polymorphic sites; when we excluded this sample, the result was non-significant (Table 2).

**Table 1:** Pairwise comparisons among lineages identified in the phylogeographic analyses of *Myiothlypis coronata*. Above diagonal: pairwise average ML corrected distances. Diagonal: average ML corrected pairwise distances between individuals within each lineage. Below diagonal: pairwise average uncorrected distances. Bold letters and numbers refer to the three mayor lineages, whereas regular letters and numbers refer to sub-populations within each mayor lineage.

	1	2	3	4	5	6	7	8	9	10	11	12	13
**1. Venezuela-Colombia (*regulus*)**	**0.021**					**0.055**					**0.068**		
2. W Venezuela (*regulus*)		0.000	0.005	0.039	0.039								
3. E slope E Andes (*regulus*)		0.005	0.001	0.036	0.036								
4. W slope E Andes (*regulus*)		0.033	0.031	0.004	0.016								
5. C and W Andes (*regulus*)		0.033	0.031	0.015	0.004								
**6. Ecuador-northern Peru**	**0.045**					**0.015**					**0.080**		
7. W Ecuador (*elata*)							0.004	0.023	0.017	0.015			
8. E Ecuador-N Peru (clade A)							0.021	0.002	0.019	0.020			
9. SW Ecuador-N Peru (clade B)							0.016	0.016	0.005	0.010			
10. NE Peru (*inaequalis*)							0.015	0.018	0.009	0.003			
**11. Central Peru-W Bolivia (*coronata*)**	**0.053**					**0.061**					**0.010**		
12. C Peru (*coronata*)												0.003	0.017
13. S Peru-W Bolivia (*coronata*)												0.015	0.004

**Table 2:** Indices of molecular diversity for lineages within *Myiothlypis coronata* based on 1041 bp ND2. Sample size (n), number of polymorphic or variable sites (var), nucleotide diversity (π), haplotype diversity (Hd), Fu's F and Tajima's D are reported. We found non-significant results for Fu's F and Tajima's D which suggest no deviations from neutrality.

Population	N	Var	π	Hd	Fu's F	Tajima's D
Venezuela-Colombia (*regulus*)	54	74	0.01821	0.869	-0.630	0.54325
Ecuador-northern Peru	75	47	0.01570	0.0156	-6.025	0.20737
W Ecuador (*elata*)	21	13	0.00242	0.922	-8.291	-1.3921
E Ecuador-N Peru (clade A)	30	15	0.00300	0.798	-5.384	-1.5543
SW Ecuador-N Peru (clade B)	15	25	0.00528	0.895	0.931	-1.2751
NE Peru (*inaequalis*)	8	9	0.00281	1.000	-5.709	-0.7656
Central Peru-W Bolivia (*coronata*)	24	30	0.00090	0.933	-7.910	0.4717

Plumage differences among Ecuadorian and northern Peruvian populations were found mainly in the ventral region (see S2 Fig for Ecuadorian specimens). Specimens of *M*. *c*. *elata* are unambiguously different from specimens identified as *M*. *c*. *orientalis*, *M*. *c*. *castaneiceps*, and *M*. *c*. *chapmani*; they have gray throat and bright yellow venter, and breast, crissum, and flaks yellow with feathers slightly emarginated with olive. All specimens were similar across localities from northern (Carchi) to southwestern Ecuador (Azuay). Individuals of *M*. *c*. *orientalis* are easily diagnosable in the northern part of its range: they have gray throat, and intermixed yellow and light-gray feathers across venter and belly, with the amount of yellow varying across localities. Specimens from Cordillera del Cóndor and Cordillera de Lagunillas (Zamora Chinchipe), Loja, and El Oro are difficult to distinguish from one another. They are mainly gray along their underparts, but the tone of gray, and the amount of yellow in the mid-belly, coppery feathers in the low venter, and yellowish coppery feathers in the crissum vary in different combinations depending on the specimen. Specimens from Piura and Cajamarca are ventrally gray. An approximation to the dominant plumage color on each sample is portrayed in Fig 1.

## Discussion

### Major lineages and barriers to dispersal

Phylogenetic and demographic analyses of ND2 revealed clear divergence and isolation of populations of *Myiothlypis coronata* in three different lineages: populations along the Andes of Venezuela and Colombia (*M*. *c*. *regulus*), populations along Ecuador-northern Peru (*M*. *c*. *elata*, *M*. *c*. *orientalis*, *M*. *c*. *castaneiceps*, and *M*. *c*. *chapmani*), and populations along central Peru-Bolivia (*M*. *c*. *coronata*). As seen in other Andean organisms (e.g. [2–4]) these major genetic breaks coincide with geographic-ecological barriers that may be responsible for the initial isolation of such populations.

Divergence between *M*. *c*. *regulus* and the Ecuador-northern Peru populations coincides geographically with the Patía and the Caquetá river valleys in southern Colombia (Fig 2). Several Andean bird species or pairs of subspecies have distributions that end abruptly around these same limits (e.g., *Cyanolyca turcosa* [41], *Aulacorhynchus derbianus* [42], *Grallaria nuchalis ruficeps* and *G*. *n*. *obsoleta* [43]). However, it seems improbable that divergence among *M*. *coronata* lineages and these other taxa was caused by geographic or ecological isolation exerted by these valleys in their present configuration. Understanding both species distributions and historical (ecological/topographic) changes in this region would be of great interest. On the other hand, population structure within *M*. *c*. *regulus* is clearly dictated by the presence of three major features: the divide of the east Andean cordillera, the Táchira Depression, and the Magdalena river valley. Limitation of gene flow across these barriers has only started to be documented with molecular data (e.g. [4, 44]), but an analysis of geographic and ecological barriers of bird populations across Colombia [45] highlights their biogeographic significance.

Separation of the Ecuador-northern Peru populations and the central Peru-west Bolivia populations (*M*. *c*. *coronata*) could be caused by the Huallaga river valley in central Peru (Fig 2). However, denser sampling around that river valley is necessary to confirm its importance in the isolation of these populations. Further south, the separation of populations of *M*. *c*. *coronata* seems to be dictated by the presence of the Apurimac river valley, which has been recognized as a potentially isolating factor between populations of other bird species (e.g. *Cyanolyca viridicyana* [46] and *Metallura tyrianthina* [47]).

### Effect of the Pleistocene on the evolution of *Myiothlypis coronata*

Our time-calibrated tree shows that most lineages have evolved during the Pleistocene (Fig 2). However, the analysis of diversification rates revealed that this pattern is consistent with a constant rate of diversification, rather than an acceleration of diversification rate during the Pleistocene (Fig 3). We were conscious that the inclusion of all the genetic lineages detected in the phylogenetic tree (Fig 2) could bias the results towards indicating a deviation from constant rates towards the mid-Pleistocene; still, the analysis favored a constant rate of diversification. Our results both agree and contrast with previous studies showing either a steady [3] or an accelerated rate of diversification of Andean birds during the Pleistocene [14, 17]. Such differences are likely to reflect the diversity of age and vagility of different taxa, as well as the diversity of evolutionary processes that promote the formation of new lineages in each case.

### Evolution of Ecuadorian and Peruvian populations

Phylogenetic analyses were unable to discern with high statistical support the sequence of splits that conduced to the four clades within the Ecuador-northern Peru lineage. Plumage changes are equally puzzling, but certain patterns emerge from the combined analysis of plumages, molecular data, and distributions. First, *M*. *c*. *elata*, which inhabits the western slope of the western Andes, is both phylogenetically and geographically separated from all other forms in Ecuador-northern Peru. It is separated from populations of *M*. *c*. *orientalis* by the Ecuadorian Andes, and seems to be restricted southwards by the Jubones river valley, where it is replaced by *M*. *c*. *castaneiceps* (clade B; Fig 4). The finding of an individual of *M*. *c*. *elata* within clade B, may be caused by current gene flow or by retention of an ancestral ND2 polymorphism, but lack of intermediate plumages in both populations favors the second hypothesis. Second, populations in clade A (including *M*. *c*. *orientalis*) inhabit the eastern slope of the east Andes, but cross the eastern Andean divide in the low areas of Loja and Zamora Chinchipe, sharing localities with individuals in clade B. Whereas both the phylogeny and the haplotype network separate these two linages clearly, gradual north-to-south change of plumage pattern of *M*. *c*. *orientalis*, from yellow-gray to virtually gray venter, suggest intergradation between *M*. *c*. *orientalis* and individuals from northern Peru currently assigned to either *M*. *c*. *castaneiceps* or *M*. *c*. *chapmani* (as suggested by Ridgely and Greenfield [13]). Third, samples of *M*. *c*. *inaequalis* form a monophyletic group geographically separated from clade B (albeit with low nodal support; Fig 1). Isolation of this population may be attributed to the presence of the Marañón river valley and the nearby North Peruvian Low, which have been regarded among the main topographic-ecological barriers to dispersal along the Andes [48, 49]. Still, phylogenetic studies have documented a diversity of responses from different taxa to the presence of these barriers, which vary from deep [8, 46, 50] to no genetic differentiation [50, 51]. In our case, only recent and shallow genetic differentiation coincides with the Marañón river valley and the North Peruvian Low.

Given the limitations of mitochondrial data in detecting hybridization, we propose the following scenario: (1) *M*. *c*. *elata*, an easily diagnosable subspecies along its range, is isolated from all other forms; (2) populations from southwestern Ecuador and northern Peru (*M*. *castaneiceps* and *M*. *chapmani*) maintain gene flow with the yellow-gray venter forms of eastern Ecuador (*M*. *c*. *orientalis*) in the southern part of its range; and (3) the northeastern Peruvian form, *M*. *c*. *inaequalis*, maintains its evolutionary independence. Unfortunately, migration rate data are not independent from sequence data, and may not reflect actual migration rates between these populations given the potential of not detecting hybridization.

### Evolution of plumage and taxonomy of *Myiothlypis coronata*

Phylogenetic and geographic analyses of *M*. *coronata* trace an evolutionary history shaped by isolation of populations across topographic-ecological barriers that may favor genetic divergence, but the degree in which such divergence is coupled with changes in plumage patterns varies across populations. For example, relatively deep genetic differentiation (0.055 substitutions per site) separates *M*. *c*. *regulus* from the Ecuador-northern Peru lineage, but only subtle plumage differences exist between *M*. *c*. *regulus* and *M*. *c*. *elata*. On the other hand, there is a striking color difference between *M*. *c*. *elata* and specimens from south Ecuador and northern Peru (clades A and B), but relatively low genetic differentiation between them (0.017 and 0.015 substitutions per site, respectively). Although our approach to analyzing plumage color change is qualitative, our results contrast with quantitative results of Winger and Bates [8], which suggest 0.032–0.054 substitutions per site as an approximate threshold for plumage differentiation among isolated bird populations across the Marañón river valley. In the case of the Russet-crowned Warbler, it would be valuable to test whether isolated populations may be responding to different selective pressures that either promote or constrain plumage evolution.

Finally, phylogeny, genetic distances, geography, and plumage pattern analyses suggest the existence of three mayor taxonomic units that qualify as Evolutionary Significant Units [10] and Unconfirmed Candidate Species [52], these are: *M*. *regulus*, *M*. *castaneiceps* (Sclater and Salvin, 1877; a polytypic species formed by populations from Ecuador-northern Peru), and *M*. *coronata*, a species with two subspecies (*M*. *c*. *coronata* and *M*. *c*. *notia*). Future efforts directed towards solving the question of whether these units qualify as independent evolutionary lineages will be of great value in establishing their taxonomic status.

## Supporting information

S1 Table Samples included in the phylogenetic and plumage analyses of *Myiothlypis coronata*. COP, Colección Ornitológica Phelps; IAvH, Instituto Alexander von Humboldt; ICN, Instituto de Ciencias Naturales; ANDES, Museo de Historia Natural, Universidad de Los Andes; QCAZ, Museo de Zoología, Pontificia Universidad Católica del Ecuador; MZUTI, Museo de Zoología, Universidad Tecnológica Indoamérica; LSUMZ, Louisiana State University, Museum of Zoology; FM, Field Museum; ANSP, Academy of Natural Sciences Philadelphia; AMNH, American Museum of Natural History.
(PDF)

S1 Fig Migrants per generation. Posterior probability distribution of the number of migrants per generation between adjacent lineages of *Myiothlypis coronata*.
(PDF)

S2 Fig Plumage variation of *Myiothlypis coronata* across Ecuador and northern Peru. *M*. *c*. *elata*: A. QCAZ 2298 (Carchi); B. QCAZ 4507 (Pichincha); C. QCAZ 4244 (Molleturo, Azuay); D. QCAZ 4111 (Cruspampa, Azuay). *M*. *c*. *orientalis*: E. QCAZ 3302 (Sumaco volcano, Napo); F. MECN 6556 (Mirador, Napo); G. MECN 4577 (Cordillera de los Huacamayos, Napo); H. MECN 4578 (Cordillera del Kutucú, Morona Santiago). Southern Ecuador: I. MECN 6690 (Cordillera del Cóndor, Zamora), MECN 7280 (Cordillera de Numbala, Loja); K. MZUTI A098 (Vilcabamba, Loja); L. MZUTI A099 (Vilcabamba, Loja); N. QCAZ 3730 (Cajanuma, Loja); M. QCAZ 4471 (Cajanuma, Loja); M. QCAZ 4473 (Cajanuma, Loja); MZUTI 77 (Salvias, El Oro).
(PDF)
