# Supplementary material for: On geographic barriers and Pleistocene glaciations: Tracing the diversification of the Russet-crowned Warbler (Myiothlypis coronata) along the Andes
Source: PLoS One. 2018 Mar 9;13(3):e0191598. doi: 10.1371/journal.pone.0191598 (PMC5844518; doi:10.1371/journal.pone.0191598)
Supplement: S1 Table — COP, Colección Ornitológica Phelps; IAvH, Instituto Alexander von Humboldt; ICN, Instituto de Ciencias Naturales; ANDES, Museo de Historia Natural, Universidad de Los Andes; QCAZ, Museo de Zoología, Pontificia Universidad Católica del Ecuador; MZUTI, Museo de Zoología, Universidad Tecnológica Indoamérica; LSUMZ, Louisiana State University, Museum of Zoology; FM, Field Museum; ANSP, Academy of Natural Sciences Philadelphia; AMNH, American Museum of Natural History. (PDF) [file pone.0191598.s001.pdf]

S1 Table. Samples included in the phylogenetic and plumage analyses of *Myiothlypis coronata*. COP, Colección Ornitológica Phelps; IAvH, Instituto Alexander von Humboldt; ICN, Instituto de Ciencias Naturales; ANDES, Museo de Historia Natural, Universidad de Los Andes; QCAZ, Museo de Zoología, Pontificia Universidad Católica del Ecuador; MZUTI, Museo de Zoología, Universidad Tecnológica Indoamérica; LSUMZ, Louisiana State University, Museum of Natural Sciences; FMNH, Field Museum; ANSP, Academy of Natural Sciences Philadelphia; AMNH, American Museum of Natural History. *Plumage analysis performed on individual. **Extracted from blood.

Tissue Number	Scientific Name	Country	State/Province	Locality	Accession number	Plumage Analysis
COP 83019	*M. c. regulus*	Venezuela	Lara	Parque Nacional Dinira	MG721211	
COP 84457	*M. c. regulus*	Venezuela	Trujillo	segundo puesto, Parque Nacional Guaramacal	MG721212	
COP 83272	*M. c. regulus*	Venezuela	Mérida	Guaraque, Portachuelo	MG721213	
COP 83273	*M. c. regulus*	Venezuela	Mérida	Guaraque, Portachuelo	MG721214	
COP 83274	*M. c. regulus*	Venezuela	Mérida	Guaraque, Portachuelo	MG721215	
COP 83275	*M. c. regulus*	Venezuela	Mérida	Guaraque, Portachuelo	MG721216	
COP 84447	*M. c. regulus*	Venezuela	Táchira	Mesa Grande, 2300m	MG721217	
COP 84448	*M. c. regulus*	Venezuela	Táchira	Mesa Grande, 2300m	MG721218	
COP 84449	*M. c. regulus*	Venezuela	Táchira	Mesa Grande	MG721219	
COP 84450	*M. c. regulus*	Venezuela	Táchira	Mesa Grande	MG721220	
COP 84451	*M. c. regulus*	Venezuela	Táchira	Mesa Grande	MG721221	
COP 84452	*M. c. regulus*	Venezuela	Táchira	Mesa Grande	MG721222	
COP 84453	*M. c. regulus*	Venezuela	Táchira	Mesa Grande	MG721223	
COP 84454	*M. c. regulus*	Venezuela	Táchira	Mesa Grande	MG721224	
COP 84455	*M. c. regulus*	Venezuela	Táchira	Mesa Grande	MG721225	
COP 84310	*M. c. regulus*	Venezuela	Táchira	El Lajón del Indio	MG721226	
COP 84456	*M. c. regulus*	Venezuela	Táchira	Sector La Barrosa	MG721227	
COP 84162	*M. c. regulus*	Venezuela	Táchira	Arriba del INIA	MG721228	
COP 84163	*M. c. regulus*	Venezuela	Táchira	Arriba del INIA	MG721229	
COP 84164	*M. c. regulus*	Venezuela	Táchira	Arriba del INIA	MG721230	
COP 83685	*M. c. regulus*	Venezuela	Táchira	via al Páramo La China	MG721231	
COP 83686	*M. c. regulus*	Venezuela	Táchira	via al Páramo La China	MG721232	
COP 83687	*M. c. regulus*	Venezuela	Táchira	via al Páramo La China	MG721233	
IAvH 11475	*M. c. regulus*	Colombia	Norte de Santander	Orocue, Sendero Arenal	MG721234	
IAvH 14805	*M. c. regulus*	Colombia	Norte de Santander	Orocue, Sendero Arenal	MG721235	
IAvH 14812	*M. c. regulus*	Colombia	Norte de Santander	Orocue, Sendero Arenal	MG721236	
IAvH 14823	*M. c. regulus*	Colombia	Norte de Santander	Orocue, Sendero Arenal	MG721237	
IAvH 14914	*M. c. regulus*	Colombia	Norte de Santander	Orocue, Sendero Arenal	MG721238	
IAvH 14922	*M. c. regulus*	Colombia	Norte de Santander	Orocue, Sendero Arenal	MG721239	
ICN 33930	*M. c. regulus*	Colombia	Norte de Santander	Orocue	MG721240	

Tissue Number	Scientific Name	Country	State/Province	Locality	Accession number	Plumage Analysis
IAvH.BT 1651	*M. c. regulus*	Colombia	Norte de Santander	Alto Río Salinas, sitio 1	MG721241	
IAvH.BT 1778	*M. c. regulus*	Colombia	Norte de Santander	Alto Río Salinas, sitio 1	MG721242	
ANDES-T 112	*M. c. regulus*	Colombia	Boyacá	SFF Iguaque, Chorrera 4, costado norte quebrada Mamarramos	MG721243	
IAvH.BT 6959	*M. c. regulus*	Colombia	Boyacá	SFF Iguaque, Chorrera 4, costado norte quebrada Mamarramos	MG721244	
ICN 35508	*M. c. regulus*	Colombia	Cundinamarca	Chicaque	MG721245	
IAvH.BT 6705	*M. c. regulus*	Colombia	Cundinamarca	La Aguadita, Fca. La Carbonera	MG721246	
IAvH.BT 6710	*M. c. regulus*	Colombia	Cundinamarca	La Aguadita, Fca. La Carbonera	MG721247	
IAvH.BT 6787	*M. c. regulus*	Colombia	Cundinamarca	Fca. San Cayetano	MG721248	
IAvH.BT 1238	*M. c. regulus*	Colombia	Huila	Camino a El Pesebre	MG721249	
IAvH.BT 1178	*M. c. regulus*	Colombia	Huila	Sendero de Andaqui a la cueva	MG721250	
IAvH.BT 7280	*M. c. regulus*	Colombia	Huila	Yalcones, El Palmar	MG721251	
IAvH.BT 7350	*M. c. regulus*	Colombia	Huila	Reserva Natural Los Yalcones	MG721252	
IAvH.BT 7382	*M. c. regulus*	Colombia	Huila	Reserva Natural Los Yalcones	MG721253	
IAvH.BT 7421	*M. c. regulus*	Colombia	Huila	El Caracol	MG721254	
IAvH.BT 7439	*M. c. regulus*	Colombia	Huila	El Caracol	MG721255	
ICN 37822	*M. c. regulus*	Colombia	Huila	Vda. Corrales, Fca. Las Nubes	MG721256	
IAvH.BT 8364	*M. c. regulus*	Colombia	Antioquia	San Sebastián de la Castellana, abajo	MG721257	
IAvH.BT 5234	*M. c. regulus*	Colombia	Antioquia	Páramo Frontino, Campo 2, El 15	MG721258	
IAvH.BT 1654	*M. c. regulus*	Colombia	Caldas	ca. 5 km S Aranzazu, Hda. Termopilas	MG721259	
IAvH.BT 1834	*M. c. regulus*	Colombia	Caldas	ca. 5 km S Aranzazu, Hda. Termopilas	MG721260	
IAvH.BT 4661	*M. c. regulus*	Colombia	Caldas	La Miel, La Cabana	MG721261	
IAvH.BT 4483	*M. c. regulus*	Colombia	Risaralda	P.M. Campoalegre, La Granja	MG721262	
IAvH.BT 2706	*M. c. regulus*	Colombia	Risaralda	PNN Tatama, La Cumbre, sitio 1	MG721263	
IAvH.BT 3983	*M. c. regulus*	Colombia	Risaralda	PNN Tatama, La Cumbre, sitio 1	MG721264	
ANSP 15539	*M. c. elata*	Ecuador	Carchi		MG721265	
ANSP 15540	*M. c. elata*	Ecuador	Carchi		MG721266	
ANSP 15762	*M. c. elata*	Ecuador	Carchi	Río La Plata, near rd. Maldonado to Tulcán	MG721267	
QCAZ 3396	*M. c. elata*	Ecuador	Carchi	Chilma Bajo	MG721268	*
QCAZ 3398	*M. c. elata*	Ecuador	Carchi	Chilma Bajo	MG721269	*
MECN 4571	*M. c. elata*	Ecuador	Carchi			*
QCAZ 3614	*M. c. elata*	Ecuador	Imbabura	San Antonio	MG721270	
QCAZ 3617	*M. c. elata*	Ecuador	Imbabura	San Antonio	MG721271	*
QCAZ 4434	*M. c. elata*	Ecuador	Imbabura	San Antonio	MG721272	
QCAZ 4435	*M. c. elata*	Ecuador	Imbabura	San Antonio	MG721273	*
QCAZ 4472	*M. c. elata*	Ecuador	Imbabura	San Antonio	MG721274	

Tissue Number	Scientific Name	Country	State/Province	Locality	Accession number	Plumage Analysis
QCAZ 4493	*M. c. elata*	Ecuador	Imbabura	San Antonio	MG721275	*
QCAZ 4508	*M. c. elata*	Ecuador	Imbabura	San Antonio	MG721276	*
QCAZ 4509	*M. c. elata*	Ecuador	Imbabura	San Antonio	MG721277	
QCAZ 4510	*M. c. elata*	Ecuador	Imbabura	San Antonio	MG721278	
MECN 4573	*M. c. elata*	Ecuador	Imbabura	Lago Cuicocha		*
QCAZ 4045	*M. c. elata*	Ecuador	Pichincha	Volcán Pululahua	MG721279	
QCAZ 4411	*M. c. elata*	Ecuador	Pichincha	Volcán Pululahua	MG721280	
QCAZ 4506	*M. c. elata*	Ecuador	Pichincha	Volcán Pululahua	MG721281	*
QCAZ 4507	*M. c. elata*	Ecuador	Pichincha	Volcán Pululahua	MG721282	*
QCAZ AV 047	*M. c. elata*	Ecuador	Pichincha	Pasochoa		*
QCAZ AV 343	*M. c. elata*	Ecuador	Pichincha	Quito		*
MECN 4570	*M. c. elata*	Ecuador	Pichincha	Chiriboga-Las Palmeras		*
MECN 4572	*M. c. elata*	Ecuador	Pichincha	-		*
MECN 4574	*M. c. elata*	Ecuador	Pichincha	Verdecocha		*
MECN 8345	*M. c. elata*	Ecuador	Cotopaxi	Río Muchipamba		*
QCAZ 3517	*M. c. elata*	Ecuador	Bolívar	Salinas	MG721283	*
QCAZ 4244	*M. c. elata*	Ecuador	Azuay	Molleturo	MG721284	*
QCAZ 4111	*M. c. elata* (clade B)	Ecuador	Azuay	Cruzpamba	MG721285	*
QCAZ 4250	*M. c. elata*	Ecuador	Azuay	Cruzpamba	MG721286	*
QCAZ 4505	*M. c. elata*	Ecuador	Azuay	Cruzpamba	MG721287	*
ANSP 19410	*M. c. orientalis* (clade A)	Ecuador	Napo	El Mirador, 12 km NNE El Chaco	MG721288	
MECN 6556	*M. c. orientalis*	Ecuador	Napo	El Mirador, 12 km NNE El Chaco		*
ANSP 19494	*M. c. orientalis* (clade A)	Ecuador	Napo	Cordillera de Guacamayos, 8 km S Cosanga	MG721289	
QCAZ 3302	*M. c. orientalis* (clade A)	Ecuador	Napo	Parque Nacional Sumaco	MG721290	*
QCAZ 3461	*M. c. orientalis* (clade A)	Ecuador	Napo	Parque Nacional Sumaco	MG721291	
QCAZ 3462	*M. c. orientalis* (clade A)	Ecuador	Napo	Parque Nacional Sumaco	MG721292	
MECN 4577	*M. c. orientalis*	Ecuador	Napo	Cordillera de los Huacamayos		*
MECN 4578	*M. c. orientalis*	Ecuador	Morona Santiago	Cordillera del Kutucú		*
ANSP 19585	*M. c. chapmani orientalis* (clade A)	Ecuador	Zamora-Chinchipe	6 km NW San Andres, N bank Río Isimanchi, E slope Cordillera Lagunillas	MG721293	
ANSP 19761	*M. c. chapmani orientalis* (clade A)	Ecuador	Zamora-Chinchipe	Cordillera del Cóndor, above Chinapinza 2000m	MG721294	
MECN 6690	*M. c. chapmani orientalis*	Ecuador	Zamora-Chinchipe	Cordillera del Cóndor		*
MECN 8104	*M. c. chapmani orientalis*	Ecuador	Zamora-Chinchipe	Cordillera del Cóndor		*
MECN 7275	*M. c. chapmani orientalis*	Ecuador	Zamora-Chinchipe	Cordillera de Numbala		*
MECN 7280	*M. c. chapmani orientalis*	Ecuador	Zamora-Chinchipe	Cordillera de Numbala		*
MECN 4579	*M. c. orientalis*	Ecuador	Loja	Cordillera de Tinajillas		*

Tissue Number	Scientific Name	Country	State/Province	Locality	Accession number	Plumage Analysis
MECN 4580	*M. c. orientalis*	Ecuador	Loja	Cordillera de Tinajillas		*
MZUTI A098**	*M. coronata* (clade A)	Ecuador	Loja	Vilcabamba	MG721295	*
MZUTI A099**	*M. coronata* (clade A)	Ecuador	Loja	Vilcabamba	MG721296	*
MZUTI A100**	*M. coronata* (clade A)	Ecuador	Loja	Vilcabamba	MG721297	*
DAPT02**	*M. coronata* (clade A)	Ecuador	Loja	Vilcabamba	MG721298	
MZUTI A104**	*M. coronata* (clade A)	Ecuador	Loja	Cerro Toledo	MG721299	
MZUTI A105**	*M. coronata* (clade A)	Ecuador	Loja	Cerro Toledo	MG721300	*
QCAZ 4482	*M. coronata* (clade A)	Ecuador	Loja	Cajanuma	MG721301	*
LSUMZ B33789	*M. coronata* (clade A)	Peru	Cajamarca	Cordillera del Cóndor, Picorana	MG721302	
LSUMZ B34716	*M. coronata* (clade A)	Peru	Cajamarca	Cordillera del Cóndor, Picorana	MG721303	
LSUMZ B31854	*M. coronata* (clade A)	Peru	Cajamarca	Quebrada Lanchal, ca. 8 km ESE Sallique	MG721304	
LSUMZ B31866	*M. coronata* (clade A)	Peru	Cajamarca	Quebrada Lanchal, ca. 8 km ESE Sallique	MG721305	
LSUMZ B31963	*M. coronata* (clade A)	Peru	Cajamarca	Quebrada Lanchal, ca. 8 km ESE Sallique	MG721306	
LSUMZ B32225	*M. coronata* (clade A)	Peru	Cajamarca	Quebrada Lanchal, ca. 8 km ESE Sallique	MG721307	
LSUMZ B32383	*M. coronata* (clade A)	Peru	Cajamarca	Quebrada Lanchal, ca. 8 km ESE Sallique	MG721308	
LSUMZ B32491	*M. coronata* (clade A)	Peru	Cajamarca	Quebrada Lanchal, ca. 8 km ESE Sallique	MG721309	
LSUMZ B32581	*M. coronata* (clade A)	Peru	Cajamarca	Quebrada Lanchal, ca. 8 km ESE Sallique	MG721310	
LSUMZ B32614	*M. coronata* (clade A)	Peru	Cajamarca	Quebrada Lanchal, ca. 8 km ESE Sallique	MG721311	
LSUMZ B33135	*M. coronata* (clade A)	Peru	Cajamarca	ca. 3 km NNE San Jose de Lourdes	MG721312	
LSUMZ B33144	*M. coronata* (clade A)	Peru	Cajamarca	ca. 3 km NNE San Jose de Lourdes	MG721313	
LSUMZ B207	*M. coronata* (clade A)	Peru	Piura	Machete on Sapalache - Carmen Trail	MG721314	
LSUMZ B211	*M. coronata* (clade A)	Peru	Piura	Machete on Sapalache - Carmen Trail	MG721315	
LSUMZ B222	*M. coronata* (clade A)	Peru	Piura	Batan on Sapalache - Carmen Trail	MG721316	
QCAZ 3730	*M. coronata* (clade B)	Ecuador	Loja	Cajanuma	MG721317	*
QCAZ 4061	*M. coronata* (clade B)	Ecuador	Loja	Cajanuma	MG721318	
QCAZ 4471	*M. coronata* (clade B)	Ecuador	Loja	Cajanuma	MG721319	*
QCAZ 4473	*M. coronata* (clade B)	Ecuador	Loja	Cajanuma	MG721320	*
MZUTI A77	*M. coronata* (clade B)	Ecuador	El Oro	vía Salvias-Tambillo	MG721321	*
LSUMZ B32272	*M. coronata* (clade B)	Peru	Cajamarca		MG721322	
LSUMZ B33781	*M. coronata* (clade B)	Peru	Cajamarca	Cordillera del Cóndor, Picorana	MG721323	
LSUMZ B33903	*M. coronata* (clade B)	Peru	Cajamarca	Cordillera del Cóndor, Picorana	MG721324	
LSUMZ B31742	*M. coronata* (clade B)	Peru	Cajamarca	Quebrada Lanchal, ca. 8 km ESE Sallique	MG721325	
LSUMZ B31767	*M. coronata* (clade B)	Peru	Cajamarca	Quebrada Lanchal, ca. 8 km ESE Sallique	MG721326	
LSUMZ B32075	*M. coronata* (clade B)	Peru	Cajamarca	Quebrada Lanchal, ca. 8 km ESE Sallique	MG721327	
LSUMZ B33069	*M. coronata* (clade B)	Peru	Cajamarca	ca. 3 km NNE San José de Lourdes	MG721328	
LSUMZ B400	*M. coronata* (clade B)	Peru	Piura	Cruz Blanca; 33 rd. km SW Huancabamba	MG721329	

Tissue Number	Scientific Name	Country	State/Province	Locality	Accession number	Plumage Analysis
LSUMZ B404	*M. coronata* (clade B)	Peru	Piura	Cruz Blanca; 33 rd. km SW Huancabamba	MG721330	
LSUMZ B417	*M. coronata* (clade B)	Peru	Piura	Cruz Blanca; 33 rd. km SW Huancabamba	MG721331	
LSUMZ B5621	*M. c. inequalis*	Peru	Amazonas	30 km by rd. E Florida on rd. to Rioja	MG721332	
LSUMZ B43484	*M. c. inequalis*	Peru	San Martín	ca. 24 km ENE Florida	MG721333	
LSUMZ B43687	*M. c. inequalis*	Peru	San Martín	ca. 24 km ENE Florida	MG721334	
LSUMZ B43708	*M. c. inequalis*	Peru	San Martín	ca. 24 km ENE Florida	MG721335	
LSUMZ B43846	*M. c. inequalis*	Peru	San Martín	ca. 24 km ENE Florida	MG721336	
LSUMZ B44043	*M. c. inequalis*	Peru	San Martín	ca. 24 km ENE Florida	MG721337	
LSUMZ B44253	*M. c. inequalis*	Peru	San Martín	ca. 22 km ENE Florida	MG721338	
LSUMZ B44435	*M. c. inequalis*	Peru	San Martín	ca. 22 km ENE Florida	MG721339	
LSUMZ B1632	*M. c. coronata*	Peru	Pasco	Santa Cruz; ca. 9 km SSE Oxapampa	MG721340	
LSUMZ B1635	*M. c. coronata*	Peru	Pasco	Santa Cruz; ca. 9 km SSE Oxapampa	MG721341	
LSUMZ B1681	*M. c. coronata*	Peru	Pasco	Santa Cruz; ca. 9 km SSE Oxapampa	MG721342	
LSUMZ B1799	*M. c. coronata*	Peru	Pasco	Santa Cruz; ca. 9 km SSE Oxapampa	MG721343	
LSUMZ B7992	*M. c. coronata*	Peru	Pasco	Playa Pampa, ca. 8 km NW Cushi on trail to Chaglla	MG721344	
LSUMZ B8014	*M. c. coronata*	Peru	Pasco	Playa Pampa, ca. 8 km NW Cushi on trail to Chaglla	JQ727427	
KUNHM 14799	*M. c. coronata*	Peru	Junín	along Río Satipo 2500	MG721345	
KUNHM 16627	*M. c. coronata*	Peru	Ayacucho	Tutumbaro	MG721346	
FMNH 398550	*M. c. coronata*	Peru	Cusco	Suecia, km 138.5 on Cusco-Shintuya Hwy, Cosnipata Valley	MG721347	
FMNH 398551	*M. c. coronata*	Peru	Cusco	Suecia, km 138.5 on Cusco-Shintuya Hwy, Cosnipata Valley	MG721348	
FMNH 398552	*M. c. coronata*	Peru	Cusco	Suecia, km 138.5 on Cusco-Shintuya Hwy, Cosnipata Valley	MG721349	
FMNH 398553	*M. c. coronata*	Peru	Cusco	Suecia, km 138.5 on Cusco-Shintuya Hwy, Cosnipata Valley	MG721350	
FMNH 398554	*M. c. coronata*	Peru	Cusco	Suecia, km 138.5 on Cusco-Shintuya Hwy, Cosnipata Valley	MG721351	
FMNH 398558	*M. c. coronata*	Peru	Cusco	Suecia, km 138.5 on Cusco-Shintuya Hwy, Cosnipata Valley	MG721352	
FMNH 398559	*M. c. coronata*	Peru	Cusco	Suecia, km 138.5 on Cusco-Shintuya Hwy, Cosnipata Valley	MG721353	
FMNH 398561	*M. c. coronata*	Peru	Cusco	Suecia, km 138.5 on Cusco-Shintuya Hwy, Cosnipata Valley	MG721354	
FMNH 430144	*M. c. coronata*	Peru	Cusco	San Pedro	MG721355	
KUMNH 18788	*M. c. coronata*	Peru	Cusco	Alto Materiato	MG721356	
AMNH.DOT 11810	*M. c. coronata*	Bolivia	La Paz	near the Río Elena	MG721357	
AMNH.DOT 11820	*M. c. coronata*	Bolivia	La Paz	near the Río Elena	MG721358	

Tissue Number	Scientific Name	Country	State/Province	Locality	Accession number	Plumage Analysis
AMNH.DOT 2700	*M. c. coronata*	Bolivia	La Paz	near the Río Elena	MG721359	
LSUMZ B22725	*M. c. coronata*	Bolivia	La Paz	Cerro Asunta Pata, 83 km by rd. E Charazani	MG721360	
LSUMZ B22808	*M. c. coronata*	Bolivia	La Paz	Cerro Asunta Pata, 83 km by rd. E Charazani	MG721361	
LSUMZ B22860	*M. c. coronata*	Bolivia	La Paz	Cerro Asunta Pata, 83 km by rd. E Charazani	MG721362	
LSUMZ B430	*M. fraseri*	Peru	Piura		MG721363	
COP 81179	*M. cinereicollis*	Venezuela	Zulia		MG721364	
IAvH-CT 470	*M. conspicillata*	Colombia	Magdalena		MG721365	
LSUMZ B11907	*M. chrysogaster*	Ecuador	Esmeraldas	El Placer	GU932058	
LSUMZ B7581	*M. bivittatus*	Venezuela	Amazonas	Cerro de la Neblina	GU932057	
LSUMZ B216	*M. nigrocristatus*	Peru	Cajamarca	Machete	GU932068	
