# Supplementary figures and images for: On geographic barriers and Pleistocene glaciations: Tracing the diversification of the Russet-crowned Warbler (Myiothlypis coronata) along the Andes
Source: PLoS One. 2018 Mar 9;13(3):e0191598. doi: 10.1371/journal.pone.0191598 (PMC5844518; doi:10.1371/journal.pone.0191598)

S1 Fig

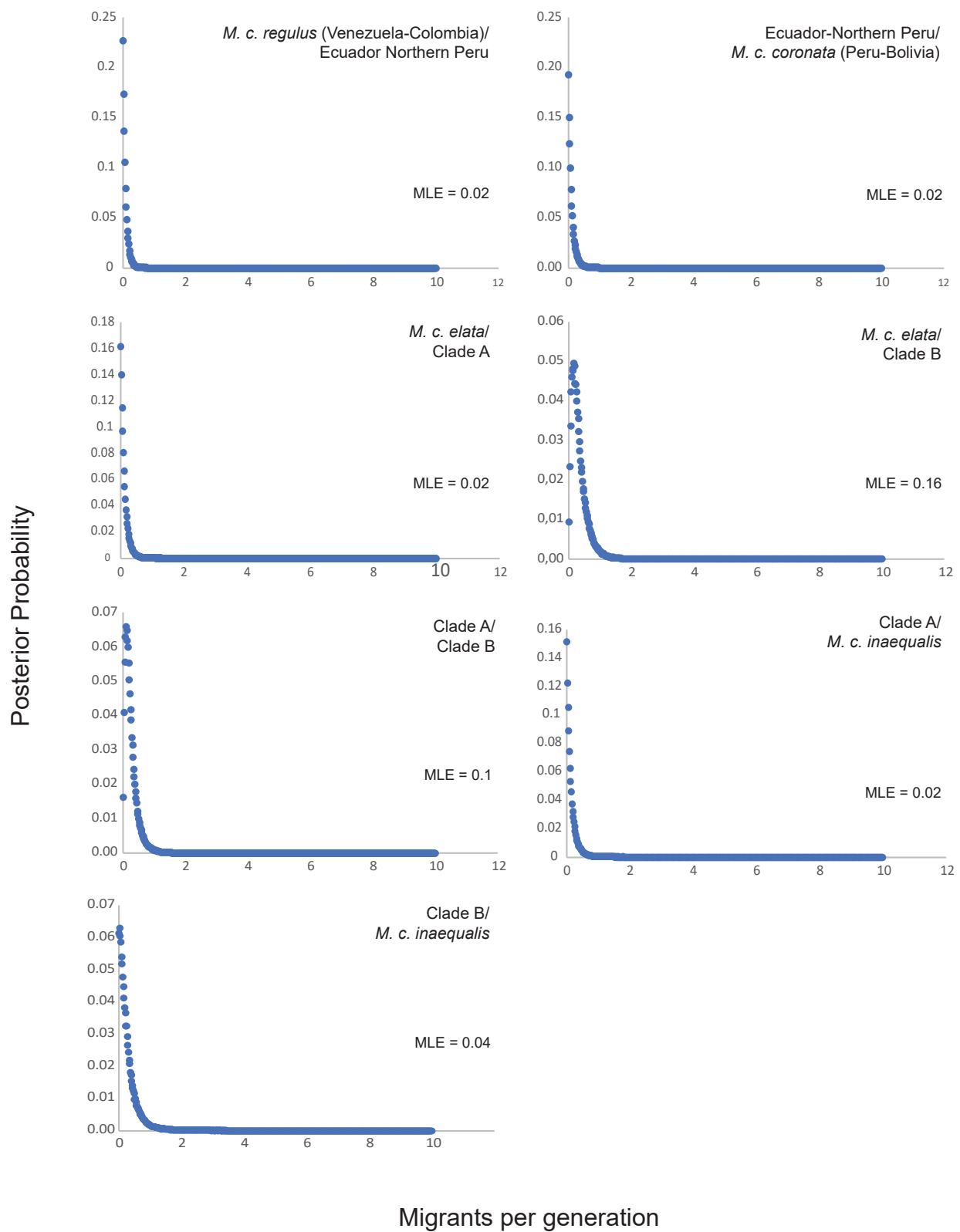

Supplement: S1 Fig — Posterior probability distribution of the number of migrants per generation between adjacent lineages of Myiothlypis coronata. (PDF) [file pone.0191598.s002.pdf]

S2 Fig

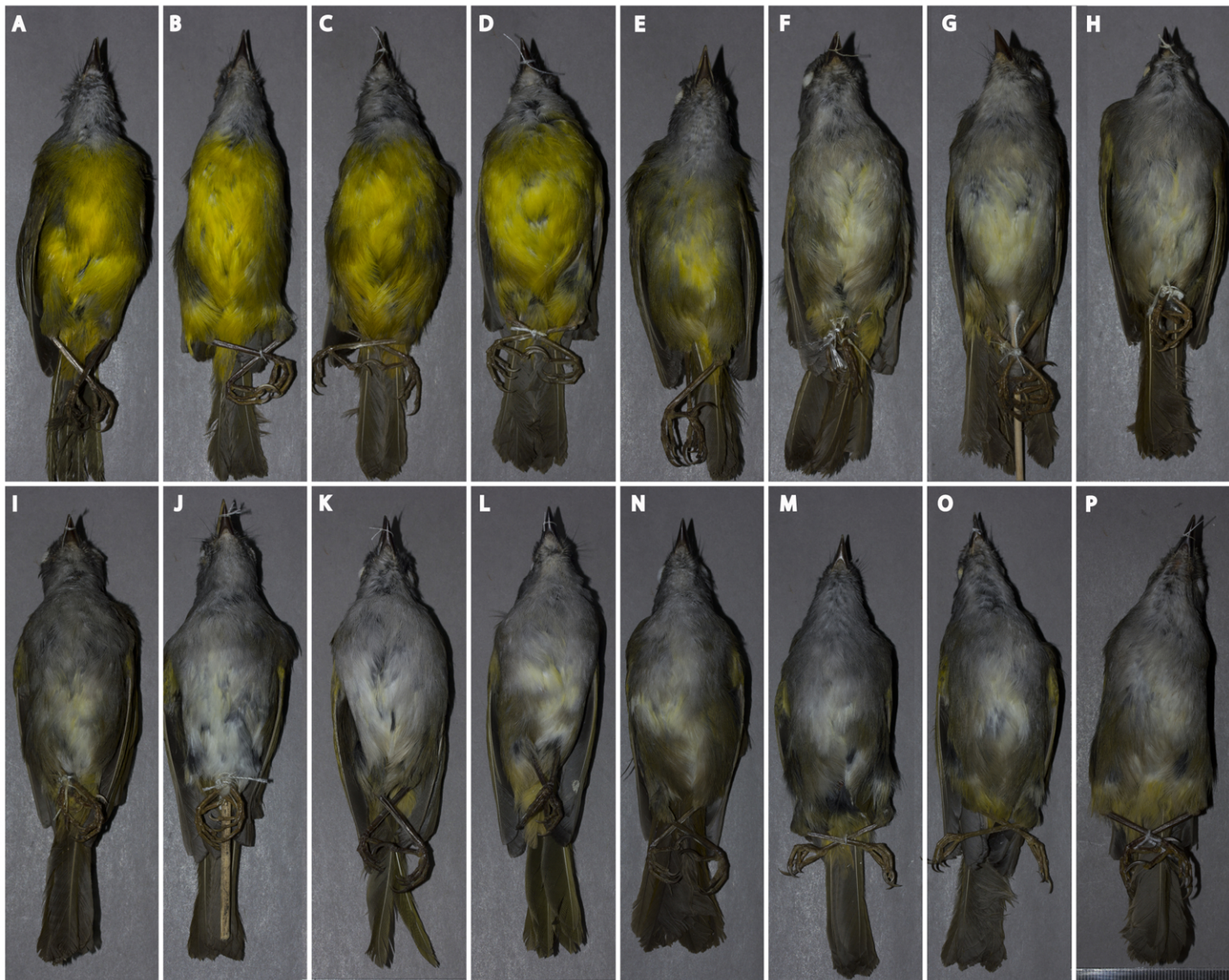

Supplement: S2 Fig — M. c. elata: A. QCAZ 2298 (Carchi); B. QCAZ 4507 (Pichincha); C. QCAZ 4244 (Molleturo, Azuay); D. QCAZ 4111 (Cruspampa, Azuay). M. c. orientalis: E. QCAZ 3302 (Sumaco volcano, Napo); F. MECN 6556 (Mirador, Napo); G. MECN 4577 (Cordillera de los Huacamayos, Napo); H. MECN 4578 (Cordillera del Kutucú, Morona Santiago). Southern Ecuador: I. MECN 6690 (Cordillera del Cóndor, Zamora), MECN 7280 (Cordillera de Numbala, Loja); K. MZUTI A098 (Vilcabamba, Loja); L. MZUTI A099 (Vilcabamba, Loja); N. QCAZ 3730 (Cajanuma, Loja); M. QCAZ 4471 (Cajanuma, Loja); M. QCAZ 4473 (Cajanuma, Loja); MZUTI 77 (Salvias, El Oro). (PDF) [file pone.0191598.s003.pdf]
